# Long-Term Retained Lippes Loop Intrauterine Device Causes Vesicouterine Fistula

**DOI:** 10.7759/cureus.38217

**Published:** 2023-04-27

**Authors:** Sean A Briceno, Matthew R Brown, Andrew B Herson, Steven T Fischer, Kevin D Healey, Brooke T Miller, Michael W. Fountain

**Affiliations:** 1 Urology, Lake Erie College of Osteopathic Medicine, Bradenton, USA; 2 Urology, AdventHealth Waterman, Tavares, USA

**Keywords:** contraception, iud device, vesico-uterine fistula, lippes loop, migrated intrauterine device

## Abstract

This case report discusses a 77-year-old female patient who presented to an outpatient clinic with urinary symptoms and recurrent UTIs. Imaging revealed a foreign body, which was later confirmed as a retained intrauterine device (IUD) that had caused a vesicouterine fistula (VUF). The patient had a medical history of cervical cancer treated with radiation therapy, during which her IUD's string could not be located, leading to the decision to proceed with radiation therapy without removing the IUD. The patient opted to manage her condition medically rather than undergo surgical removal due to concerns about worsening the vesicouterine fistula. This case highlights the potential risks and complications of retained IUDs, and the importance of careful consideration and communication among clinical teams and patients when managing these situations.

## Introduction

In the United States, intrauterine devices (IUDs) have been available since the 1960s, with options including non-medicated, hormonal, and copper products [1]. The utilization of long-acting reversible contraceptives (LARCs) has been on the rise since the early 2000s [2]. As of 2020, there were approximately 100 million women with IUDs globally [3]. With the United States having an unintended pregnancy rate of around 50% [4], it is crucial to provide a greater range of safe and effective contraceptive options, including long-acting reversible contraceptives (LARCs) such as intrauterine devices (IUDs). In underdeveloped countries, IUDs constitute 16.5% of the birth control methods used, while in developed countries, they account for 9.4% [5]. Due to a lack of adequate health education among some women, when they experience symptoms related to an IUD's inflammatory reaction, they may not be able to promptly identify and receive a diagnosis for the foreign body [3], thus leading to urology-related diseases. 

The most effective forms of reversible contraception are long-acting and reversible methods (LARC), such as IUDs and the etonogestrel subdermal contraceptive implant [6]. With an overall failure rate of less than 1% in the first year of use, the IUD is considered one of the most effective forms of contraception [1]. The use of IUDs and implants is associated with the highest rates of contraceptive continuation among women who opt for these methods [6]. There are two primary types of modern IUDs: copper and hormonal. Copper IUDs elicit a localized inflammatory response that increases the release of prostaglandins, rendering the uterus unsuitable for implantation [7]. Moreover, copper ions have a toxic effect on sperm, impeding their movement beyond the cervix [7]. Conversely, levonorgestrel (LNG) IUDs change the texture of cervical mucus, causing it to thicken and impede the progress of sperm while simultaneously reducing the thickness of the endometrium, rendering the uterus unsuitable for implantation [7]. 

IUD insertion is typically a straightforward and safe gynecological procedure. The failure rate for a levonorgestrel-releasing IUD is around 0.2%, while copper-containing IUDs have a failure rate of approximately 0.8%, with typical use [4]. While complications are rare, they can be severe, including uterine perforation and loss of the device. The reported incidence of uterine perforation is between 0.05 and 13 per 1000 insertions, and the results can be serious. If an IUD migrates into surrounding organs, such as the bladder or sigmoid colon, it can cause further complications. Lost IUDs can be located and retrieved using minimally invasive procedures like hysteroscopy, endoscopy, and laparoscopy [4]. Due to the anatomical proximity of the uterus to the bladder, the insertion of an IUD can cause harm to the uterus [3]. When the bladder is full and contracts, it comes into close contact with the front wall of the uterus, which can lead to damage. If such damage occurs, the IUD may shift to the bladder through the injured area during uterine contractions and become lodged there as a foreign object [3]. In today’s literature, there have been roughly 70 cases of IUDs migrating to the bladder, which in turn have caused complications such as stone formation, infections, and bladder perforation [8]. We report a case of a retained IUD that subsequently caused a vesicouterine fistula (VUF).

## Case presentation

A 77-year-old female with a past medical history of hypertension, type 2 diabetes mellitus, cervical cancer treated with radiation therapy, and a surgical history of a complicated colonoscopy and laparotomy due to adhesions presented to our outpatient clinic with complaints of urinary urgency, frequency, and recurrent UTIs. She reported that her urinary urgency and frequency had been present for the past six months. She denied any fever or burning with urination. The patient’s physical exam was unremarkable. For the prior six months, she had been treated for multiple UTIs with ciprofloxacin and nitrofurantoin. Initial vital signs on presentation were all within normal limits. Laboratory tests included a complete blood count (CBC), a complete metabolic panel (CMP), a urine dipstick, and a urine culture. The CBC was unremarkable. The CMP showed a low estimated glomerular filtration rate (eGFR), elevated blood urea nitrogen (BUN), calcium, and creatinine (Table 1).

**Table 1:** Complete metabolic panel. BUN: blood urea nitrogen; eGFR: estimated glomerular filtration rate; A/G ratio: albumin to globulin ratio; mg/dL: milligrams per deciliter; mL/min/1.73 m^2^: milliliters per minute per 1.73 meters squared; mmol/L: millimoles per liter; g/dL: grams per deciliter; U/L: units per liter.

	Laboratory value	Reference range
BUN	31 mg/dL	7-25 mg/dL
Creatinine	1.26 mg/dL	0.7-1.5 mg/dL
eGFR	44 mL/min/1.73 m^2^	>60 mL/min/1.73 m^2^
BUN/creatinine ratio	25	6-22
Sodium	140 mmol/L	135-145 mmol/L
Potassium	4.7 mmol/L	3.5-5 mmol/L
Chloride	105 mmol/L	95-105 mmol/L
Carbon dioxide	23 mmol/L	22-32 mmol/L
Calcium	10.6 mg/dL	8.5-10.5 mg/dL
Protein, total	6.5 g/dL	6.5-8.1 g/dL
Albumin	4.1 g/dL	3.5-5.0 g/dL
Globulin	2.4 g/dL	2.0-3.5 g/dL
A/G ratio	1.7 g/dL	0.8-2.0 g/dL
Bilirubin, total	0.6 mg/dL	0.0-1.2 mg/dL
Alkaline phosphatase	70 U/L	25-125 U/L
Aspartate transaminase	25 U/L	10-35 U/L
Alanine transaminase	23 U/L	0-31 U/L

The urine dipstick came back positive for nitrates and a small amount of leukocyte esterase (Table 2), indicative of a UTI. The urine culture showed greater than 100,000 CFU/mL of extended-spectrum beta-lactamases (ESBL) in *Escherichia coli*.

**Table 2:** Urine dipstick. pH: potential hydrogen; POC: point of care.

	Laboratory value	Reference range
Source-urine	Urine, clean catch	
Color-urine	Yellow	
Clarity-urine	Cloudy	Clear
pH-urine	5.5	5.0-7.0
Protein-urine	Negative	Negative
POC glucose-urine	Negative	Negative
Ketones-urine	Negative	Negative
Blood-urine	Small	Negative
Nitrite-urine	Positive	Negative
Leukocyte esterase-urine	Small	Negative

**Figure 1:**
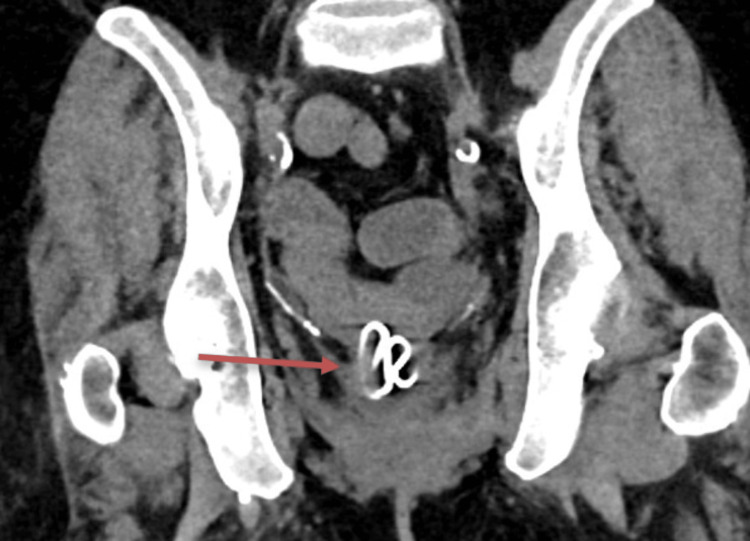
A computerized tomography urogram (CTU) demonstrating a retained Lippes loop device (red arrow) inside the patient's posterior inferior bladder. No bladder leak was identified by the radiologist.

A computerized tomography urogram (CTU) was ordered and revealed bilateral simple renal cysts. After reviewing the images with the urology team and the radiologists, a foreign body was discovered in the posterior bladder wall (Figure 1).

**Figure 2:**
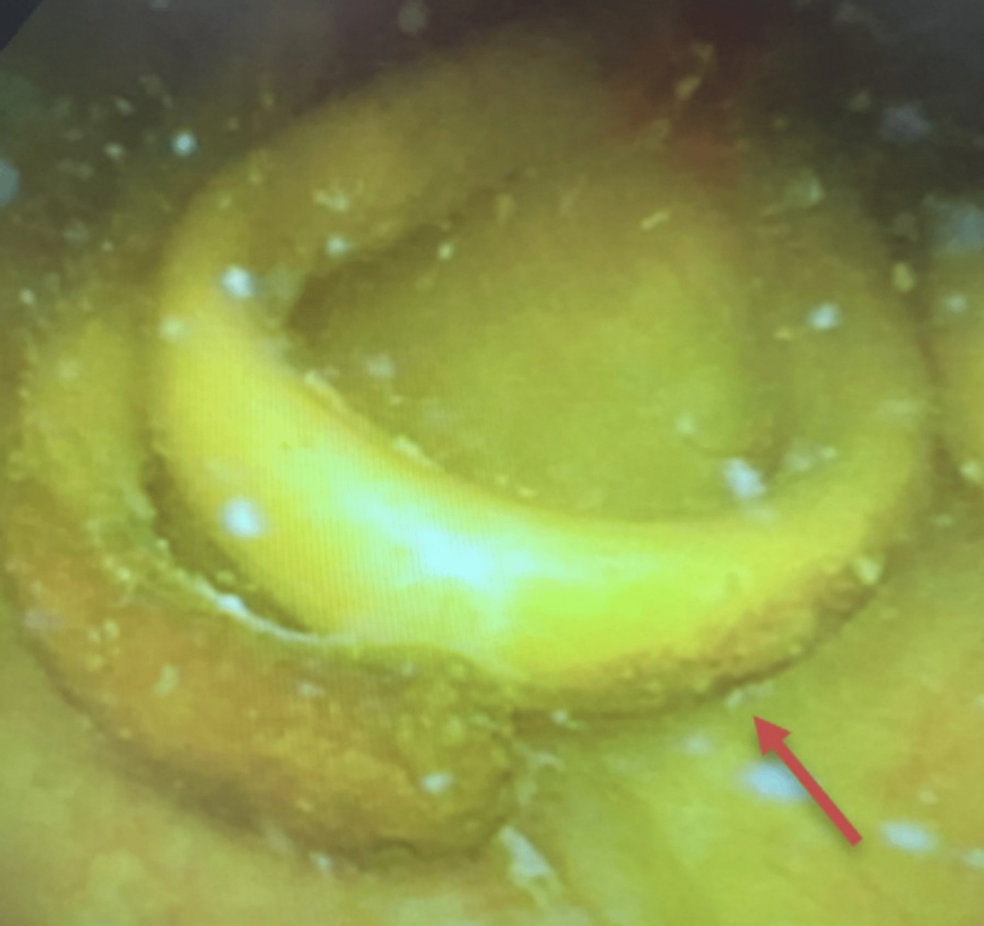
A cystoscopy of the patient’s bladder demonstrating a retained Lippes loop device (red arrow) within the posterior bladder wall.

The case was discussed with two radiologists and an obstetrics/gynecology physician. After multiple conversations with colleagues, the foreign body was determined to possibly be a Lippes loop intrauterine device (IUD). A cystoscopy was performed and revealed the same foreign body eroding through the posterior bladder wall and confirmed the identity of the foreign body as a Lippes loops IUD (Figure 2), creating a vesicouterine fistula. 

Upon further questioning of the patient and presentation of the results, she revealed that she was aware of the retained IUD. In the 1990s, the patient underwent radiation therapy for her cervical cancer. It was mentioned that her IUD would need to be taken out prior to the administration of her radiation; however, the radiation team could not locate the IUD’s string. At that time, the patient and her radiation team decided to proceed with the radiation therapy without the removal of the IUD. The results of the cystoscopy were discussed with the patient as well as the concern that the removal of the IUD could possibly worsen her vesicouterine fistula. The patient was informed of other options: laparoscopic or open surgical approaches to remove the IUD. Considering the patient's prior surgical experiences involving complex laparotomies due to adhesions, the patient was advised that surgical intervention may be intricate and result in prolonged surgery and anesthesia. The potential drawbacks of extended surgery/anesthesia, such as the potential impact on the patient's quality of life, were also thoroughly discussed with the patient. Considering the clinical findings and the patient's history of radiation treatment, the care team and the patient jointly agreed to retain the IUD in its current position and pursue medical treatment instead of surgical removal. The patient expressed that the risks and recovery associated with a surgical procedure outweighed the burden of recurrent UTIs. The patient was prescribed a regimen of trimethoprim-sulfamethoxazole and was advised to schedule a follow-up appointment in six months for a repeat cystoscopy to assess the possibility of further erosion. At that time, the urological team and the patient may re-evaluate the treatment plan based on the findings.

## Discussion

A vesicouterine fistula (VUF) is a rare medical condition where an abnormal connection forms between the urinary bladder and the uterus or cervix [9]. This condition typically occurs as a result of an iatrogenic injury during a cesarean section, accounting for the majority of cases [9]. VUF accounts for 1-4% of all reported urogenital fistulas [10]. The presence of a vesicouterine fistula may result in various complications, such as infection, bacteremia, infertility, abortion, and other related issues [10]. VUF typically presents with urinary incontinence, cyclic hematuria, and amenorrhea [11].

IUDs are one of the most common and effective pregnancy prevention options [8,12]. In the US, 10.4% of women use some form of long-acting reversible contraception, including IUDs as well as other implantable contraceptives [12]. In comparison, 14% use birth control pills, 18.1% use female sterilization, and 5.6% use male sterilization as contraceptive methods [12]. Globally, IUDs are the most commonly used reversible contraceptive method, estimated to have over 150 million users, and are only second to female sterilization for overall contraception [7]. When used for five years, they are shown to be >98% effective at preventing pregnancy [7]. Modern IUDs come in two main forms: copper or hormonal. Copper IUDs cause a local inflammatory reaction that leads to increased prostaglandin release, making the uterus inhospitable for implantation [7]. The copper ions are toxic to spermatozoa, preventing their progression past the cervix [7]. Levonorgestrel (LNG) IUDs alter the consistency of the cervical mucus, thickening it, preventing superior progression of sperm, as well as thinning the endometrium, making the uterus inhospitable [7]. 

Modern IUD options come in a similar form, T-shaped plastic containing barium for imaging purposes, with strings that attach to the inferior pole of the device, which allow for monitoring of placement and for removal. Previously, the Lippes loop was a type of intrauterine device (IUD) used for contraception. It was first introduced in the 1960s by Dr. Jack Lippes, a gynecologist who worked at the University of Buffalo [13,14]. The Lippes loop IUD was a flexible polyethylene plastic double “S” loop that composed an overall trapezoidal shape. This shape was formulated to better fit the natural shape of the uterine cavity to decrease the incidence of device expulsions. While the Lippes loop was popular for a time, it fell out of favor in the 1970s and 1980s due to reports of complications, including pelvic infections and perforation of the uterus [13,14]. 

The occurrence rate of uterine perforation due to IUD insertion is estimated to range between 1.3 and 1.6 cases per 1000 insertions, highlighting that perforation is a relatively rare but potentially severe complication [5]. Such perforations may take place either immediately due to improper insertion or years later as a result of device migration [5]. One rare complication of IUDs is bladder perforation. Research suggests that bladder perforation generally occurs soon after insertion of the IUD. Other contributing factors to bladder perforation are retroverted uteruses, multiparity, recent abortions, C-sections, lactation, and sepsis [5]. Some signs of abnormally placed IUDs are pain, cramping, irregular bleeding, dyspareunia, and absent IUD strings [5]. The guidelines for locating a misplaced IUD are to obtain a transvaginal and transabdominal ultrasound. The initial modality for examining IUD migration, particularly in cases involving stone formation due to IUDs, is an abdominal X-ray [5]. A pelvic CT scan can be used as well. Bladder perforation can be suspected when a patient with a misplaced IUD presents with urinary symptoms [15]. In the case presented, the patient was complaining of frequent UTIs, and the device was directly visualized using a cystoscopy instead of radiographically. The patient's radiation therapy likely contributed to the bladder perforation as the radiation therapy likely produced scarring, which helped facilitate a perforation into the bladder wall.

The treatment for a retained IUD and bladder perforation is with various types of surgery, depending on the location of the IUD [4]. Previously, open laparotomies and laparoscopic removals have been successfully performed on IUDs that have perforated through the uterus [4]. Regarding the patient presented in this case, the decision was made to leave the IUD in place, as removal of the IUD either intact or in fragments could potentially lead to a worsening of her vesicouterine fistula, potentially decreasing the patient’s quality of life. Laparoscopic and/or open surgery was considered; however, these were ultimately not performed due to the patient’s history of adhesion and the possibility of decreasing her quality of life.

## Conclusions

Our case report highlights a compelling scenario in which recurrent UTIs led to the identification of a vesicouterine fistula caused by a retained IUD. While IUDs are effective at providing long-term contraception for women, our patient's situation demonstrates that they can also result in severe complications, such as recurrent UTIs and vesicouterine fistula formation. Computerized tomography urogram (CTU) and cystoscopy revealed a retained Lippes loop IUD that migrated to the patient’s bladder. Surgical removal is a standard treatment, but our patient opted for medical treatment due to concerns about risks and recovery. Adequate health education is essential for women regarding IUDs and their potential complications and available treatment options.
